# Fish mouths as engineering structures for vortical cross-step filtration

**DOI:** 10.1038/ncomms11092

**Published:** 2016-03-29

**Authors:** S. Laurie Sanderson, Erin Roberts, Jillian Lineburg, Hannah Brooks

**Affiliations:** 1 Department of Biology, College of William and Mary, PO Box 8795, Williamsburg, Virginia 23187, USA

## Abstract

Suspension-feeding fishes such as goldfish and whale sharks retain prey without clogging their oral filters, whereas clogging is a major expense in industrial crossflow filtration of beer, dairy foods and biotechnology products. Fishes' abilities to retain particles that are smaller than the pore size of the gill-raker filter, including extraction of particles despite large holes in the filter, also remain unexplained. Here we show that unexplored combinations of engineering structures (backward-facing steps forming *d*-type ribs on the porous surface of a cone) cause fluid dynamic phenomena distinct from current biological and industrial filter operations. This vortical cross-step filtration model prevents clogging and explains the transport of tiny concentrated particles to the oesophagus using a hydrodynamic tongue. Mass transfer caused by vortices along *d*-type ribs in crossflow is applicable to filter-feeding duck beak lamellae and whale baleen plates, as well as the fluid mechanics of ventilation at fish gill filaments.

## Abstract (web summary)

Suspension-feeding fish, such as goldfish and whale sharks, retain prey without clogging their oral filter. Using a cross-step filtration model based on fish morphology, Sanderson *et al*. show how vortices generated by this design could trap and transport particles even smaller than the filter pores.

## Graphical abstract

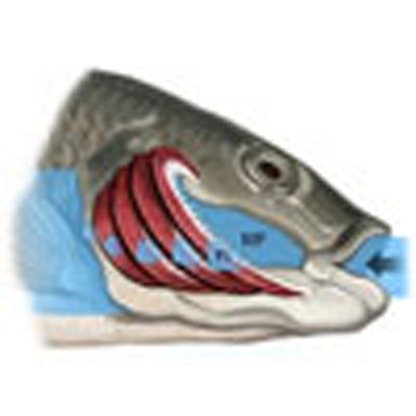

Although more than 70 species of suspension-feeding fishes compose 25% of the world fish catch, the fluid dynamic processes enabling fish to avoid clogging of the gill-raker filter are unknown. Not only does their gill-raker filter remain free from particle accumulation, suspension-feeding fishes are also able to retain and concentrate particles that are smaller than the filter pores and can even retain particles when large regions of the gill-raker filter are absent^1^,^2^,^3^,^4^,^5^. In addition to the ecological and evolutionary relevance, these problems are of substantial interest to industrial filtration engineers who seek to reduce the major operating expenses associated with clogging^6^.

Suspension-feeding fishes had been assumed to use dead-end mechanical sieving, in which fluid passes perpendicularly through the filter, whereas particles that are too large to exit through the pores are retained by sieving on the filter surface^7^. In contrast, crossflow filtration in suspension-feeding fish species has been shown recently to extract food particles without clogging or concentration polarization along the rows of comb-like, mesh-like or knobby gill rakers that form the filter surface on the branchial arches^1^,^7^,^8^,^9^,^10^ (Fig. 1a). However, the inertial lift forces employed in microfluidics devices are too low to account for the lack of contact between food particles and the gill-raker filter^1^, indicating that additional unidentified mechanisms are operating to prevent clogging during crossflow filtration in fishes.

Our three-dimensional (3D) physical models of oral cavities tested in a recirculating flow tank demonstrate a new crossflow process, termed cross-step filtration, that achieves particle retention and transport without clogging. Previous theoretical and experimental filtration models have not considered the three-dimensionality of branchial arches and the slots between arches^11^,^12^,^13^,^14^. These structures create *d*-type roughness, characterized by a series of grooves between closely spaced spanwise ribs that form backward-facing steps along a tube or channel, with a groove aspect ratio (groove width *w* divided by rib height *h*) of <3 to 4 (refs 15, 16). Inertial microfluidics devices for cell collection^17^ have converged on nonporous grooves (trapping chambers or expansion-contraction reservoirs) that essentially create *d*-type roughness. Unlike the *k*-type roughness (groove aspect ratio >∼4) used frequently in industrial heat exchanger and filtration designs, *d*-type roughness is uncommon in fluid engineering applications because the interaction of mainstream flow with closely spaced *d*-type ribs generates a sustained concentrated vortex that extends fully across the nonporous groove between consecutive ribs^15^,^16^.

Paddlefish (*Polyodon spathula*) and basking sharks (*Cetorhinus maximus*) are distant phylogenetically, but both are ram suspension feeders that swim forward with a fully open mouth while retaining zooplankton (0.2–4.0 mm) as water exits between the gill rakers^18^,^19^ (Fig. 1b,c). Their branchial arches are convergent, having a novel rib-and-groove arrangement in which mainstream flow within the oral cavity is separated from the porous gill rakers by deep slots between the branchial arches (Fig. 1b–d). We refer to the grooves between arches as slots in these species to highlight that gill rakers form the porous exterior (lateral) aspect, through which filtrate exits. The *d*-type ribs embedded in the walls of our physical models, mimicking the branchial arches, are a series of backward-facing steps, with the slot between two consecutive ribs acting as a temporary downstream expansion of the model diameter. Across a vast range of Reynolds numbers (Re) from 10^−4^ to 10^5^ (Re=*Uhv*^−1^, *U*=mainstream velocity, *h*=step height=rib height, *v*=kinematic viscosity), far broader than the size range from juvenile to adult suspension-feeding fishes, a backward-facing step with *h*≥∼100 μm generates a recirculation region directly downstream of the step in a nonporous tube or channel^20^,^21^,^22^.

Given the ubiquity of disruptive vortices caused by backward-facing steps in industrial fluid mechanical systems ranging from miniature blood pumps to jet engines and power plant gas turbines, considerable experimental effort focuses on attempts to minimize the extent of the recirculation region and control the separated flow downstream of backward-facing steps^23^,^24^. Numerical simulation of this complex 3D recirculation continues to be challenging, especially with particulate flows^25^, and the location of the vortices precludes visualization using fiberoptic endoscopy or digital particle image velocimetry in the 3D slots between ribs, making physical modelling a valuable alternative^26^,^27^. Our cross-step filtration model based on paddlefish and basking shark morphology takes advantage of vortical flow in porous slots to reduce clogging by concentrating particles along the slot margins. Furthermore, by varying model parameters, we show that modified configurations can generate vortices that suspend and transport concentrated particles.

## Results

### Particle retention in physical models with *d*-type ribs

During suspension feeding, the branchial arches of paddlefish and basking sharks form *d*-type ribs with a groove aspect ratio (groove width *w* divided by rib height *h*) ranging from ∼1.2 to 2 (Table 1). Our physical models had *d*-type ribs with a groove aspect ratio ranging from 0.9 to 1.6. As fish have muscular control of branchial arch abduction, we designed models with *d*-type ribs angled at 110°, 90° or 55° along the midline of the oral cavity roof (Fig. 2, flow parameters in Table 2).

Owing to the models' conical shape, the rib that was downstream of each slot diverted a portion of the mainstream flow into zone 1. This flow that was closest to the downstream rib exited from the slot almost perpendicularly through the mesh in zone 1, depositing some particles there (Fig. 2). The rib that was directly upstream of each slot generated a recirculation region continuously in zone 2 (Fig. 3a, Table 2 and Supplementary Movie 1). In addition, flow that travelled directly over each rib inside the model then separated from the downstream corner edge of the rib to form a shear layer that wrapped around the recirculation region in zone 2 (Fig. 3b). Inertial particles accumulated in zone 3 where some of the vortical flow from the shear layer exited through the mesh (Fig. 2).

Branchial arch angle affected the pattern of particle deposition on the mesh in zones 1 and 3 (Fig. 2) but did not significantly affect the total mass of particles retained by the models with different rib angles (Fig. 4; one-way analysis of variance (ANOVA); *P*=0.48; *n*=5 models for each angle). Notably, the mesh in zone 2 of all slots remained particle-free as particles were transported by the posterior-to-anterior vortical flow of the shear layer that encircled the recirculation region.

### Cross-step filtration with an incomplete mesh

Particles were concentrated in zones 1 and 3 of the cross-step models wherever mesh was present, even where mesh covered only a single slot or a small portion of a slot (Fig. 3c). Removal of the mesh from an entire posterior slot (7.0% of total mesh area; Fig. 5a,b) caused a reduction of only 10.2% in the dry mass of particles retained by the 90° cross-step model. We compared this cross-step model with a standard crossflow model that lacked ribs and slots but had identical dimensions for the gape, total mesh area and mesh pore size. Removal of 2.8% of the mesh from the standard crossflow model (Fig. 5c,d) caused a significant reduction of 21.0% in the dry mass of particles retained (Fig. 5e; two-way ANOVA with Tukey's *post-hoc* test; *P*<0.0001; *n*=5 models for each design).

### Control of axial direction of vortex travel

The recirculation region encircled by the separated shear layer composed a vortex that was generated continuously in zone 2 of all slots as water flowed over the ribs inside all cross-step models. In the basic cross-step model design (Fig. 2), these vortices were shed in bursts out of the model through the mesh at irregular time intervals and unpredictable locations along the slots. The addition of an asymmetrical transparent plastic skirt around the anterior portion of the model simulated a fish operculum (gill cover) or elasmobranch gill flap to control the axial direction of vortex travel inside the slots (Fig. 3a,b and Supplementary Movie 1). The skirt limited the exit of water from the roof of the model more than from the model's floor, passively creating a sink near the floor. This sink trapped the vortices inside the slots, similar to suction orifices in the lateral walls of a nonporous channel that can trap vortices downstream from *d*-type ribs^28^, and caused fluid to travel along the vortex axis in zones 2 and 3 towards the model floor. For the 90° cross-step model with the simulated operculum and a mainstream flow speed inside the model of 10.1±0.1 cm s^−1^, the average maximum speed of the recirculation region along its axis in the spanwise slot was 3.0±0.3 cm s^−1^ (mean±s.d., *n*=5 vortices, Table 2).

### Vortex linear speed and model pressure

The linear speed of the vortex in the recirculation region was comparable to the mainstream flow speed. For the 90° cross-step model with the simulated operculum and a mainstream flow speed inside the model of 10.1±0.1 cm s^−1^, the average maximum linear flow speed of the recirculation region was 9.7±0.6 cm s^−1^ (mean±s.d., *n*=5 vortices, Table 2). The pressure inside this 90° cross-step model was 11.5±4.2 Pa above ambient (mean±s.d., *n*=5 models).

### Particle retention and transport in suspension-feeding fish

When paddlefish preserved in suspension-feeding position were placed in the flow tank, vortical flow (Fig. 6a) caused particle concentration in zone 3 between the branchial arches (Fig. 6b), matching model functioning. Suspension-feeding fishes close their mouth at intervals of ∼2–30 s and generate water currents inside the oral cavity, apparently transporting retained particles into position for swallowing^18^,^19^,^29^. To create a potential ‘hydrodynamic tongue'^19^,^30^,^31^ for particle transport in the models, a continuous nonporous barrier of polymer film was placed on the mesh in zone 3 of each slot. When combined with the addition of a simulated operculum and alterations of rib and slot parameters, the nonporous barrier on the mesh in zone 3 caused the trapped vortex^28^ in each slot to lift particles from the mesh and transport suspended particles within the slots (Supplementary Movie 2). This nonporous barrier in zone 3 simulated the band of mucus-covered muscle and elastic fibres along the slot margins in paddlefish and basking sharks^32^,^33^.

## Discussion

Cross-step filtration is a unique fluid dynamic process that concentrates and transports particles by integrating all four major components of the 3D architecture in fish oral cavities: (i) branchial arches that are backward-facing steps forming *d*-type ribs and slots attached to the (ii) porous gill raker surfaces of (iii) the conical oral cavity covered by (iv) an operculum or elasmobranch gill flap that directs the axial travel of the vortices within the slots. This filtration mechanism is dependent on the presence of crossflow that was identified by Sanderson *et al*.^1^ using computational fluid dynamics and fiberoptic endoscopy in three phylogenetically diverse families of suspension-feeding fishes. The mainstream flow through our models and preserved paddlefish is an anterior-to-posterior crossflow that is tangential to the *d*-type ribs formed by the branchial arches. As the crossflow travels posteriorly in the conical oral cavity, the interaction of the crossflow with the branchial arch that is directly upstream of each slot generates a spanwise vortex in that slot. The use of *d*-type ribs with a groove aspect ratio (groove width *w* divided by rib height *h*) of <3 to 4 (refs 15, 16) causes the vortex to affect flow across the entire width of the slot. This cross-step filtration design is highly adaptable, with particle concentration and transport determined by vortex parameters, which in turn are affected by structural parameters of the cone, *d*-type ribs, slots and mesh.

**Figure 1:**
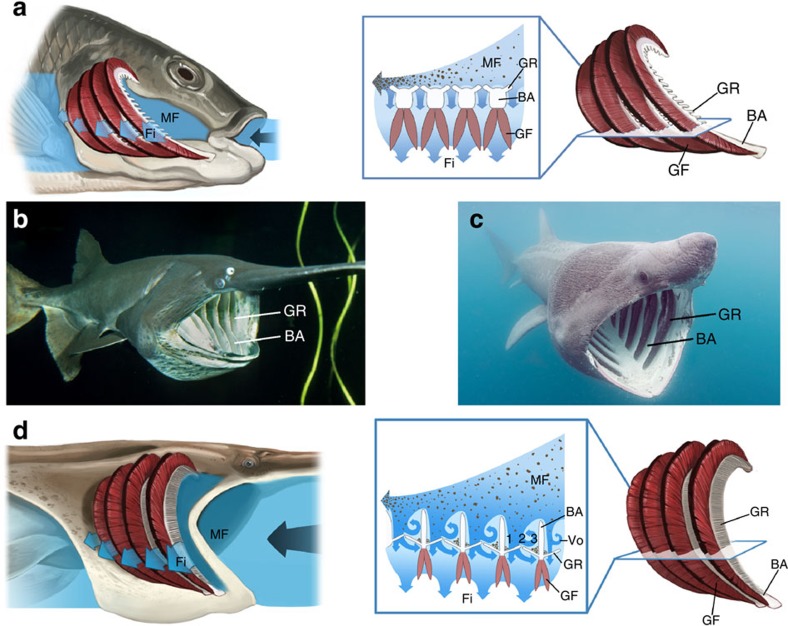
Filtration models in suspension-feeding fishes. (**a**) Current model of crossflow filtration. Mainstream flow travels tangentially across the branchial arches and concentrates particles in the posterior oral cavity^1^. Filtrate exits between gill rakers and passes the gill filaments where gas exchange occurs. (**b**) Paddlefish with gill rakers forming the porous floor of deep slots between branchial arches. (**c**) Convergent morphology in the basking shark. (**d**) Vortical cross-step filtration model. Mainstream flow interacts with the series of backward-facing steps formed by the branchial arches. The resulting vortical flow interacts with the gill rakers to concentrate particles in zones 1 and 3 along the slot margins. BA, branchial arch; Fi, filtrate; GF, gill filament; GR, gill raker; MF, mainstream flow; Vo, vortex. This figure is not covered by the CC BY licence. [**a**,**d** © Virginia Greene/virginiagreeneillustration.com; **b**, © Kevin Schafer/kevinschafer.com; **c**, © Doug Perrine/SeaPics.com.] All rights reserved, used with permission.

**Figure 2:**
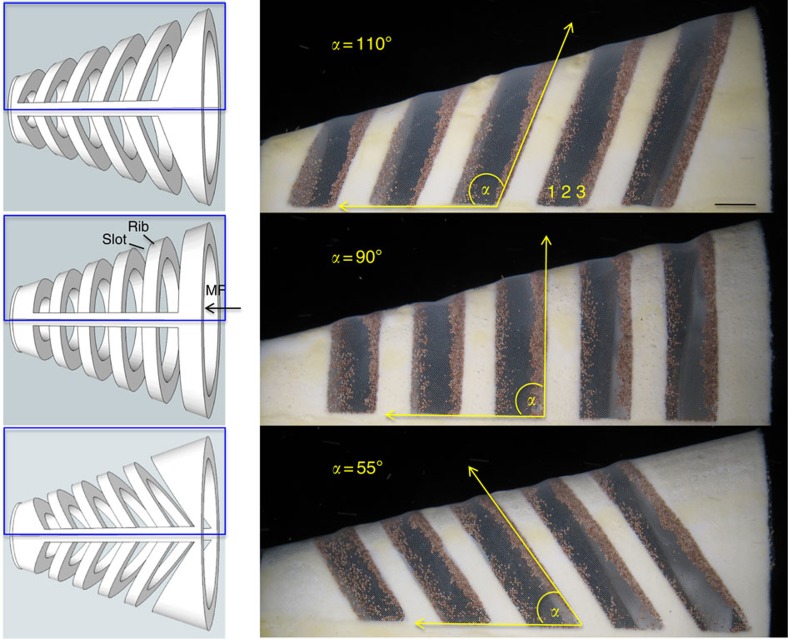
The concentration of particles by backward-facing steps that form *d*-type ribs in 3D models. (Left) Computer-aided design (CAD) images of 3D models, top view. Mainstream flow from the right entered the gape of the model; filtrate exited via the slots between ribs. (Right) Enlarged view of 3D-printed models (from blue boxes on left) with 140- μm mesh covering exterior of slots. *Artemia* cysts (brine shrimp eggs,∼250 μm diameter) were concentrated in zones 1 and 3 of all slots, whereas vortical flow within each slot prevented clogging in zone 2. MF, mainstream flow. Scale bar, 0.5 cm.

Vortical cross-step filtration can incorporate multiple filtration modes that are illustrated in Figs 1d and 2: anterior-to-posterior crossflow (MF) above backward-facing steps, dead-end mechanical sieving (zone 1), posterior-to-anterior vortical crossflow (zone 2), concentration of inertial particles (zone 3) and particle suspension and transport in vortices (zones 2 and 3, Supplementary Movie 2). In our models and in the preserved paddlefish, a porous mesh formed the gill-raker filter along the floor of the slots. The rib that was downstream of each slot diverted some mainstream flow directly through this mesh in zone 1, causing particle deposition by dead-end mechanical sieving in zone 1 (Figs 1d and 2). During cell concentration using inertial microfluidics, forces induce cells to migrate laterally across streamlines into nonporous grooves^34^,^35^. In contrast, in our models and the preserved paddlefish, particles were carried into zone 1 and around the periphery of the vortex in zone 2 by water that passed over the backward-facing step, entered the slot and subsequently exited through the mesh.

**Figure 3:**
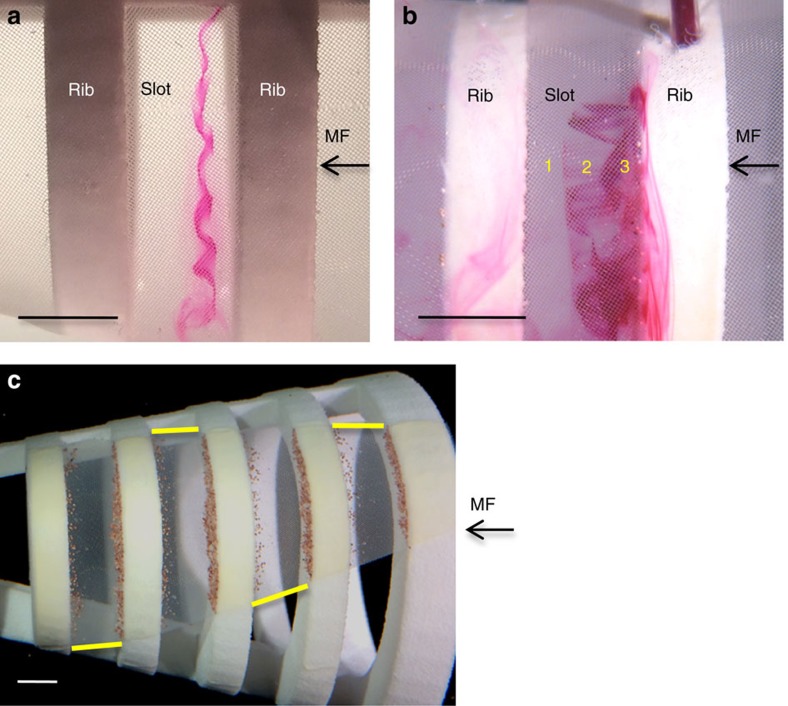
Vortices enable effective cross-step filtration with an incomplete mesh. (**a**) A recirculation region was generated in each slot by the backward-facing steps of all cross-step models, visualized here using dye travelling in a spanwise direction after being released through the tip of a cannula that was flush with the slot wall at the top of the model (top view). (**b**) The separated shear layer entered the slot and wrapped around the recirculation region in each slot (side view). The shear layer was visualized here using dye released from a cannula inserted through a rib (top right of image). This dye tracked the flow that passed immediately over the rib inside the model, where the tip of the cannula was flush with the rib wall. (**c**) Even when mesh was missing from the entire left side of a model, the posterior slot, and the top and bottom of all other slots on the right side, the vortex formed by the recirculation region and the shear layer concentrated particles along the slot margins wherever mesh was present (side view). Yellow lines drawn in alternate slots delineate extent of mesh. Scale bars, 0.5 cm. MF, mainstream flow.

**Figure 4:**
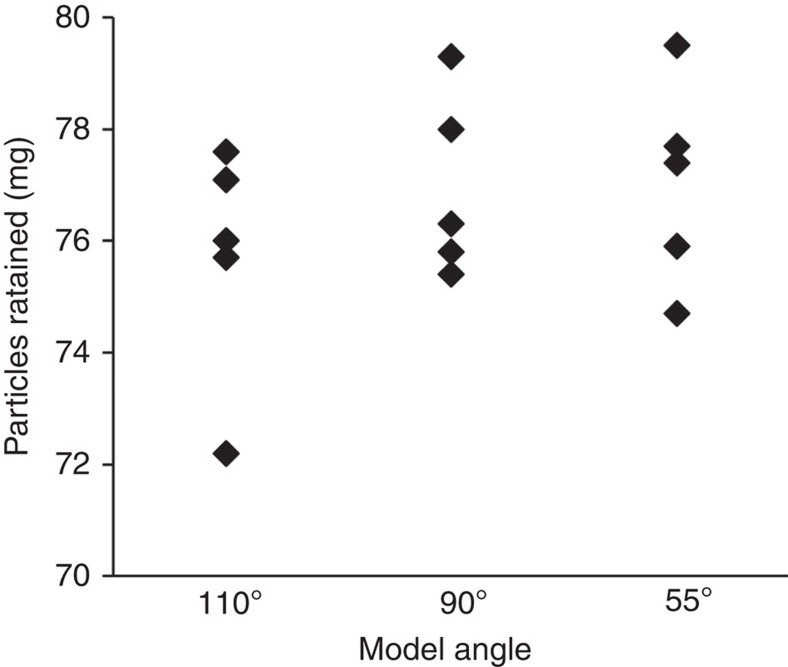
Branchial arch angle did not significantly affect the total mass of particles retained by cross-step models. Mass of *Artemia* cysts retained by models with branchial arches angled at 110°, 90° or 55° along the midline of the oral cavity roof (one-way ANOVA; *P*=0.48; *n*=5 models for each angle).

In zone 2, vortical crossflow from posterior to anterior along the mesh within the slot prevented clogging of the mesh. Industrial engineers improve crossflow filtration using designs that create high shear rates to clear the filter surface^6^,^36^. In our models, the downstream corner edge of each backward-facing step caused a separated shear layer to form from the water that passed directly over the rib and entered the slot. This shear layer wrapped around the recirculation region in zone 2, causing a high shear rate that enhanced filtration (Fig. 3a,b). By configuring backward-facing steps to form *d*-type ribs on a porous surface, the cross-step models created persistent vortices in zone 2 that were sustained as water entered the slot and exited through the mesh. Suction along the bottom of the downstream wall in a backward-facing step has been used as an active control technique to reduce the disruptive effects of flow separation and recirculation in engineering applications with nonporous channels^37^,^38^. In contrast, our cross-step design harnessed the separated shear layer and the recirculation region to prevent clogging of the porous mesh during filtration. As the mainstream flow that was immediately overlying the separated shear layer also entered the slot, the linear flow speed of the separated shear layer and the recirculation region that were closest to the mesh (9.7±0.6 cm s^−1^, Table 2) were comparable to that of the mainstream flow (10.1±0.1 cm s^−1^).

The vortical crossflow inside the slot transported inertial particles into zone 3, where they were deposited (Figs 1d,2 and 6). In suspension-feeding fishes, these vortices could also cause particles that are smaller than the pore size of the mesh to encounter sticky oral surfaces by direct interception or inertial impaction^39^,^40^. Particle retention in mucus strings or aggregates on the branchial arches and gill rakers has been recorded endoscopically in suspension-feeding Nile tilapia (*Oreochromis niloticus*, Cichlidae)^41^. Mucus has been calculated to compose ∼12% of the epibranchial organ content and 10% of the foregut content by dry mass in suspension-feeding gizzard shad (*Dorosoma cepedianum*, Clupeidae)^42^, indicating the potential importance of mucus for particle retention in suspension-feeding fishes.

As additions to the models, we designed (i) an asymmetrical external skirt that simulated an operculum or elasmobranch gill flap and (ii) a solid strip of polymer film that simulated the tissue and mucus layer along the bases of the gill raker rows in paddlefish and basking sharks. The external skirt allowed more water to exit from the bottom (ventral) side of the model than from the top (dorsal) side, which manipulated the recirculation region in zone 2 to become a stable trapped vortex^28^,^43^ that travelled axially along the slot towards the bottom of the model (Supplementary Movie 1). A solid polymer strip covering the external surface of the mesh along the slot margin in zone 3 prevented water from exiting out of zone 3 and resulted in the suspension, concentration and transport of particles in the vortex along zones 2 and 3 (Supplementary Movie 2). In this manner, inertial particles remained suspended in both the shear layer and the recirculation region, preventing particle deposition in zone 3.

Our cross-step models created a ‘hydrodynamic tongue'^19^,^30^,^31^ that controlled the axial direction of vortex travel along the modified solid margins of the slots. The structural modifications could be adjusted to suspend and transport concentrated particles as needed, potentially including particles that are smaller than the pore size of the mesh. Thus, suspension-feeding fishes could fine-tune these functional morphological features to control the axial direction of vortex travel and thereby manipulate the transport of concentrated particles that are either in suspension or bound in shear-thinning mucus strands. For example, using this mechanism, suspension-feeding fishes could transport concentrated particles in suspension to the ceratobranchial-epibranchial junctions at the corners of the oral cavity, which are parallel to the entrance of the oesophagus.

Particles that are smaller than the mesh size could remain suspended and be transported in the vortical crossflow that travels axially along the slot^44^. Smith and Sanderson^2^ quantified significant ingestion of polystyrene microspheres (11–200 μm diameter) during suspension feeding in the tilapia species *Oreochromis aureus* and *O. esculentus* (Cichlidae) following surgical removal of the entire gill-raker filter and all microbranchiospines from the branchial arches. Fiberoptic endoscopy in these freely swimming fish confirmed that mucus strings or aggregates were not present on the branchial arches during feeding^45^. Early studies based on industrial crossflow filtration designs have suggested vortices as a potential mechanism in suspension-feeding fishes for controlling the suspension and transport of small particles, thereby preventing fouling of the filter surface^1^,^11^,^46^,^47^.

**Figure 5:**
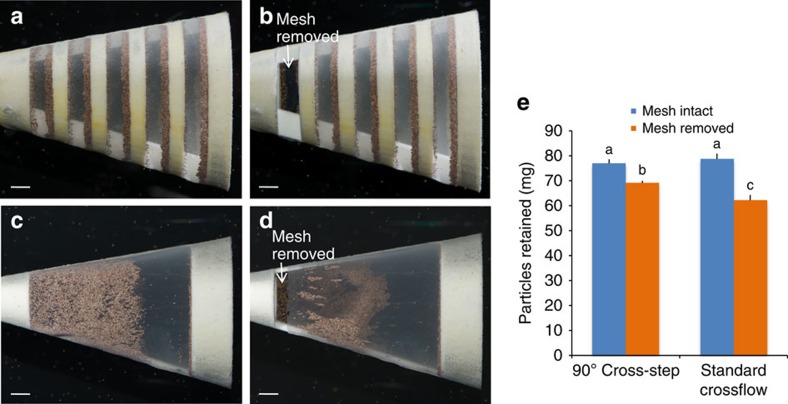
Particle retention after removal of mesh from cross-step model compared with standard crossflow model. (**a**,**b**) Removal of mesh from the right posterior slot (7.0% of total mesh area) in the 90° cross-step model had minimal effect on particle retention in the other slots (side view). (**c**,**d**) Removal of 2.8% of the posterior mesh from the right side of a standard crossflow model lacking ribs and slots caused a significant reduction in particle retention (side view). (**e**) Particle retention with mesh intact versus removed (mean±s.d., *n*=5 models for each design). Means with different letters indicate *P*<0.0001 (two-way ANOVA with Tukey's *post-hoc* test). Photos **a**–**d** by P. Yañez. MF, direction of mainstream flow inside model. Scale bars, 0.5 cm.

**Figure 6:**
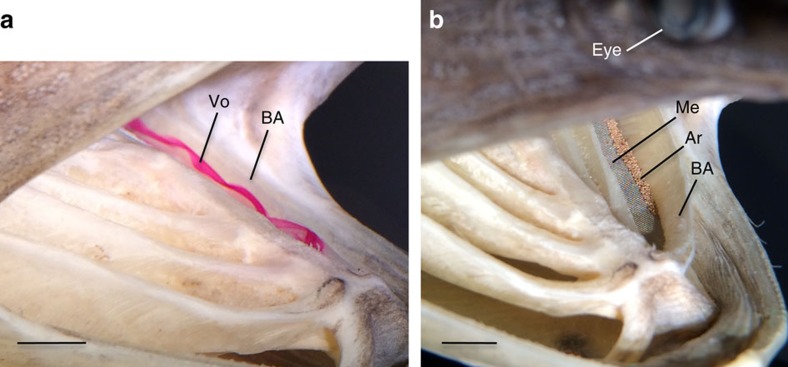
Vortex generation and particle concentration in paddlefish preserved in suspension-feeding position. (**a**) Tracer dye visualization of vortex generated directly downstream of the backward-facing step formed by the first branchial arch on the fish's left side. (**b**) The vortex concentrated *Artemia* cysts on the mesh primarily in zone 3 of the slot, directly downstream of the first branchial arch. Ar, *Artemia* cysts; BA, branchial arch; Me, mesh; Vo, vortex. Scale bars, 0.5 cm.

Effective operation of our cross-step models despite large holes in the mesh (Figs 3c and 5) is facilitated by the extremely small pressures involved. The pressure inside the 90° cross-step models with intact mesh was 11.5±4.2 Pa (mean±s.d., *n*=5 models) above ambient, comparable to the small oral pressures recorded in paddlefish during ram ventilation^4^. Industrial crossflow filtration is performed at transmembrane pressures that are 1–4 orders of magnitude higher^48^. The surprising performance of the cross-step filter despite an incomplete mesh is consistent with previously unexplained reports of basking shark and young paddlefish suspension feeding with only partially developed gill rakers^4^,^5^.

Given that vortices are nearly universal behind backward-facing steps in the presence of crossflow^20^,^21^,^22^, *d*-type ribs could have significant unrecognized impacts on biological function in aquatic and aerial flow. In our cross-step models, flow retained a strong vortical movement after exiting between the ribs. This turbulence could deliver water to fish gill filaments, particularly those located near zone 3 (Figs 1d and 3b). As more than 30,000 fish species possess branchial arches that may form *d*-type ribs, potential vortex formation in the slots between branchial arches has substantial implications for the fluid dynamics of fish feeding and ventilation throughout ontogeny and evolution. Vortical cross-step filtration could be applicable to feeding in a diversity of fish species. In addition, many filtration structures involved in vertebrate suspension feeding are composed of *d*-type ribs in crossflow, including fish gill rakers, tadpole gill filters, bird beak lamellae and whale baleen plates, suggesting that principles of vortical cross-step filtration could have widespread application.

## Methods

### Design of cross-step physical models

Cross-step physical models based on anatomical descriptions, measurements and observations of paddlefish and basking sharks^18^,^19^,^32^,^33^,^49^,^50^,^51^,^52^ were designed using SketchUp Pro 2014 (Trimble Navigation) and 3D-printed in nylon plastic (fine polyamide PA 2200, Shapeways). The cartilaginous branchial arches of both species are elongated in a dorsal–ventral direction to form slots as deep as 7–10 cm between successive arches in paddlefish (>2 m maximum body length^50^,^51^,^52^) and basking sharks (7 m maximum body length^33^). The branchial arches form portions of the walls along the oral floor and roof. Thus, the height of the backward-facing steps (rib height, *h*) formed by the branchial arches in our cross-step models was equal to the thickness of the model wall in which the steps were embedded, which graded gradually from 3.7 mm at the oral roof to 6.7 mm at the oral floor. The anterior to posterior width of the slot between branchial arches in our models (groove width, *w*) varied from 6.1 to 6.9 mm, creating *d*-type ribs with *wh*^−1^ ranging from 0.9 to 1.6.

The exceptionally long and slender gill rakers (keratinous in basking sharks^49^; ossified in paddlefish^50^) are attached to the branchial arch walls along the exterior (lateral) surfaces of the deep slots^33^,^50^ rather than attaching to the interior (medial) surfaces of the branchial arches. During ram suspension feeding in both species, muscles at the bases of the comb-like gill rakers contract to extend the rakers across the bottom of each slot between consecutive branchial arches^18^,^32^,^33^. Therefore, we glued nylon mesh (140-μm pore size, 55% open area, Component Supply Co.) to the exterior of the models to cover the slots.

Cross-step models with branchial arches angled at 110°, 90° or 55° along the midline of the oral cavity roof were designed with identical gape area and cone dimensions. These three designs varied by <1.3% in the medial area of the slot openings. The total open pore area of the slots was 160% of the area of the models' gape.

### Flow tank experiments

For each replicate (*n*=5 models for each angle), we mounted the model in the centre of a recirculating flow tank^53^ (18 × 18 × 90 cm working area, 100 l total volume) using a sting attached to the closed downstream end of the model. The flow tank was seeded with 0.6000, g brine shrimp cysts (*Artemia* sp., 210–300 μm diameter, density 1.09 g cm^−3^, 10 p.p.m. volume concentration) and the model retained particles for 3.0 min at a flow tank speed of 18.4±0.4 cm s^−1^ (mean±s.d., flow parameters in Table 2). After covering the model gape and removing the model from the flow tank, we used prefiltered water to rinse *Artemia* cysts from the model over a Nalgene 310-4000 filter holder fitted with a tared Nalgene 205-4045 membrane filter (0.45-μm pore size). The retained *Artemia* cysts were dried to constant mass and weighed to the nearest 0.0001, g (Fisher Scientific XA-100 analytical balance).

### Comparison with standard crossflow model

For comparison with the cross-step models, a standard crossflow model was designed, 3D printed and covered in 140-μm mesh as above (*n*=5 models). This model had a wall thickness of 1.5 mm and lacked ribs and slots. The dimensions for the gape and the total mesh area were identical to the 90° cross-step model. We then used digital calipers and fine dissecting scissors to prepare modified crossflow models (*n*=5 models) and modified 90° cross-step models (*n*=5 models) by removing a section of the posterior mesh to create a hole on the right side of each model. We conducted flow tank experiments as described above, with the addition of a flexible silicone sheet (McKeon Products) that covered the posterior hole in the mesh while each model was removed from the flow tank.

### Modified cross-step model designs

Additional cross-step models were equipped with an asymmetrical external plastic skirt around the anterior portion of the model. The external skirt simulated a fish operculum (gill cover) or elasmobranch gill flap to control the axial direction of vortex travel inside the slots, and was transparent to allow vortex visualization. In combination with the external skirt, some models were configured with a continuous nonporous barrier of clear polymer film over zone 3 in each slot. As the basic cross-step model design is highly flexible, experiments were also conducted using modifications of rib and slot parameters (for example, V-shaped versus U-shaped slots).

### Flow speed measurement

A miniature flow probe was constructed from a glass bead thermistor (1.09 mm diameter, 112-101BAJ-01, Fenwal Electronics) and connected to a circuit modified from LaBarbera and Vogel^47^,^54^. A Sonometrics TRX-4A/D convertor sampled the output of the circuit at 200 Hz. Values from the A/D convertor were confirmed using digitized recordings of rhodamine water-tracing dye (Cole Parmer) released anterior to the model (125 frames per s, Intensified Imager VSG, Kodak). To measure the mainstream flow speed entering the gape of the 90° model, the flow probe was threaded through a polyethylene cannula (1.57 mm inner diameter (ID), 2.08 mm outer diameter (OD), Intramedic PE-205) that was placed directly in front of the model. To measure the flow speed in the interior of the 90° model, the polyethylene cannula was inserted through a hole (2.38 mm diameter) drilled in the top of the model and was placed flush with the interior model surface. The glass bead was protruded slightly from the tubing until a flow speed maximum was recorded.

We visualized flow inside the models using rhodamine dye introduced slowly through a polyethylene cannula (1.14 mm ID, 1.57 mm OD, Intramedic PE-160) by gravity feed or a syringe. The cannula was inserted into holes (1.59 mm diameter) drilled through the walls of the model. By placing the tip of the cannula flush against the interior surface of the model wall, we ensured that the tubing did not generate a wake.

### Pressure measurement

We used a Millar Mikro-tip SPC-330 catheter pressure transducer (1.0 mm diameter) connected to a PCA-2 preamplifier and calibration unit and Sonometrics TRX-4A/D convertor. The transducer was threaded through a water-filled polyethylene cannula (1.14 mm ID, 1.57 mm OD, Intramedic PE-160) inserted through a 1.59-mm hole drilled in the top of the 90° models. Flexible caulk sealed the distal end of the cannula. To measure total pressure, the sensor area faced upstream, perpendicular to the mainstream flow. When the flow tank motor was turned on, the stable pressure increase inside the model was due to the static and dynamic components.

### Paddlefish specimens

Juvenile paddlefish (36.6–50.5 cm total length, 19.8–29.0 cm eye-to-fork length, *n*=3 fish) were obtained from Big Fish Farms (William and Mary Institutional Animal Care and Use Committee approval 07/30/14; Virginia Department of Game and Inland Fisheries approval 07/24/14). Specimens were received on ice within 24 h of death and were preserved in 10% buffered formalin with their mouth and branchial arches manipulated to be in suspension-feeding position^19^. When paddlefish and basking sharks are not feeding or are dead, elastic fibres passively pull the gill rakers to lie flat against the walls of the branchial arches. The gill rakers rest vertically against the branchial arch walls until abductor muscles that are attached to the gill rakers contract during feeding^33^,^50^. Thus, the gill rakers of the preserved paddlefish did not extend across the slot between adjacent branchial arches. To simulate the gill rakers, we cut stainless steel mesh (104 μm pore size, Ted Pella Inc.) to fit inside the slot between the first two branchial arches along the lateral margins of the slot where the gill rakers originate.

We mounted preserved paddlefish in the flow tank using an overhead clamp that was flush with the body surface located posterior to the gill cover. We inserted an infusion needle (25G x ¾”) through the downstream ventral gill cover into the first branchial arch, with the needle tip placed flush against the downstream wall of the branchial arch. At a flow tank speed of 18 cm s^−1^, vortical flow along the slot downstream of the first branchial arch was traced using rhodamine dye released slowly from the infusion needle by gravity feed or a syringe. The flow tank speed was at the low end of the range reported for swimming speeds in live paddlefish of approximately the same size as these preserved paddlefish^19^. We released *Artemia* sp. cysts into the flow tank anterior to the paddlefish gape.

### Statistical analysis

We used JMP 12 Mac (SAS Institute Inc.) at a level of significance of *P*<0.05 for all statistical tests. Levene's tests for homogeneity of variance and Shapiro–Wilk tests for normality were performed. We compared the mass of *Artemia* cysts retained by models designed with rib angles of 110°, 90° or 55° using a one-way ANOVA. To compare the performance of the standard crossflow model and the 90° cross-step model with mesh intact versus removed, we used a two-way ANOVA followed by Tukey's honest significant difference (HSD) *post-hoc* test.

## Additional information

**How to cite this article**: Sanderson, S. L. *et al*. Fish mouths as engineering structures for vortical cross-step filtration. *Nat. Commun.* 7:11092 doi: 10.1038/ncomms11092 (2016).

## Supplementary Material

Supplementary Movie 1 Trapped vortex in 90° cross-step model with external transparent plastic skirt. Mainstream flow (10 cm s^-1^) entered from gape at right (top view, 120 frames s^-1^). The asymmetrical skirt caused fluid to travel along the trapped vortex axis in zones 2 and 3 towards the model floor where the exit of water from the slots was less restricted by the shape of the skirt. In this movie, rhodamine dye travels in a spanwise direction after being released through a cannula that is flush with the top wall of the slot. The vortex inside the slot originates at the top of the model, travels away from the viewer down the side of the model, and then can be seen exiting from the slot when the dye reaches the bottom of the model. Ruler in mm.

Supplementary Movie 2 Modifications in cross-step model parameters that mimic suspension-feeding fishes caused transport of concentrated particles along the vortex axis within each slot. Mainstream flow (25 cm s^-1^) entered from gape at right (side view, 30 frames s^-1^ ). Here, modifications of rib and slot parameters combined with (1) a continuous nonporous barrier of clear polymer film covering a portion of the mesh in zone 3 of each slot and (2) a transparent external skirt around the anterior region of the model created a stable trapped vortex in each slot that lifted Artemia cysts from the mesh and transported them towards the floor of the model. A posterior margin of the external skirt is visible at lower left.

## Figures and Tables

**Table 1:** Calculations of groove aspect ratio (*wh* ^−1^) for slots between the branchial arches during suspension feeding by fish species for which data are available in the literature.

Species	Groove width *w*	Rib height *h*	Groove aspect ratio (*wh*^−1^)	Source
*Polyodon spathula* (Polyodontidae, paddlefish)	0.37–0.79 cm(*n*=3 fish)	0.38–0.50 cm(*n*=3)	1.2±0.3(mean±s.d., *n*=3)	This study
*Cetorhinus maximus*(Cetorhinidae, basking shark)	20 cm	10 cm	2	Data in Matthews and Parker^33^
*Oreochromis aureus* (Cichlidae, blue tilapia)	0.5 mm	1.4 mm	0.4	Figures and videos in Smith and Sanderson^2^,^47^

**Table 2:** Flow parameters for 90° cross-step model.

*Flow speed (mean±s.d., n=5 models)*	
Flow tank (no model)	18.4±0.4 cm s^−1^
3 cm anterior of model gape	12.6±0.2 cm s^−1^
Immediately anterior of model gape	10.5±0.3 cm s^−1^
Mainstream flow inside centre of model	10.1±0.1 cm s^−1^
	
*Reynolds number (dimensionless)*	
Gape (4.0 cm hydraulic diameter)	4,200
Backward-facing step (0.6 cm height)	605
Particle (250 μm diameter)	25
Mesh pore (140 μm diameter)	14
	
*Recirculation region inside slot (average maximum values with simulated operculum attached; mean±s.d., n=5 vortices)*	
Diameter	0.30±0.02 cm
Linear speed	9.7±0.6 cm s^−1^
Speed along axis in spanwise slot	3.0±0.3 cm s^−1^
Rotational speed	626±68 revolutions per min
